# Ferritin Assembly in Enterocytes of *Drosophila melanogaster*

**DOI:** 10.3390/ijms17020027

**Published:** 2016-02-05

**Authors:** Abraham Rosas-Arellano, Johana Vásquez-Procopio, Alexis Gambis, Liisa M. Blowes, Hermann Steller, Bertrand Mollereau, Fanis Missirlis

**Affiliations:** 1 Department of Physiology, Biophysics and Neuroscience, Cinvestav del IPN, Avenida IPN 2508, Zacatenco, Mexico City 07360, Mexico; rosas.arellano@gmail.com (A.R.-A.); yo-ana3@hotmail.com (J.V.-P.); 2 Howard Hughes Medical Institute, Strang Laboratory of Apoptosis and Cancer, The Rockefeller University, New York, NY 10065, USA; agambis@gmail.com (A.G.); steller@mail.rockefeller.edu (H.S.); 3 Department of Organismal Biology, School of Biological and Chemical Sciences, Queen Mary, University of London, Mile End Road, London E1 4NS, UK; l.m.blowes@qmul.ac.uk; 4 Laboratory of Molecular Biology of the Cell, UMR5239 CNRS/Ecole Normale Supérieure de Lyon, IFR 128 Biosciences Lyon Gerland, Université de Lyon, Lyon 69007, France; Bertrand.Mollereau@ens-lyon.fr

**Keywords:** biosynthesis, complex formation, confocal microscopy, enterocyte, feedback control, insect, iron, metal, midgut, vesicular traffic

## Abstract

Ferritins are protein nanocages that accumulate inside their cavity thousands of oxidized iron atoms bound to oxygen and phosphates. Both characteristic types of eukaryotic ferritin subunits are present in secreted ferritins from insects, but here dimers between Ferritin 1 Heavy Chain Homolog (Fer1HCH) and Ferritin 2 Light Chain Homolog (Fer2LCH) are further stabilized by disulfide-bridge in the 24-subunit complex. We addressed ferritin assembly and iron loading *in vivo* using novel transgenic strains of *Drosophila melanogaster*. We concentrated on the intestine, where the ferritin induction process can be controlled experimentally by dietary iron manipulation. We showed that the expression pattern of *Fer2LCH-Gal4* lines recapitulated iron-dependent endogenous expression of the ferritin subunits and used these lines to drive expression from *UAS-mCherry-Fer2LCH* transgenes. We found that the Gal4-mediated induction of mCherry-Fer2LCH subunits was too slow to effectively introduce them into newly formed ferritin complexes. Endogenous Fer2LCH and Fer1HCH assembled and stored excess dietary iron, instead. In contrast, when flies were genetically manipulated to co-express Fer2LCH and mCherry-Fer2LCH simultaneously, both subunits were incorporated with Fer1HCH in iron-loaded ferritin complexes. Our study provides fresh evidence that, in insects, ferritin assembly and iron loading *in vivo* are tightly regulated.

## 1. Introduction

With over one million insect species on earth [1], there can be no simple generalized description of the iron storage strategies they employ [2,3,4,5,6,7,8,9,10,11]. Nevertheless, insect ferritins are widely recognized as the key protein complexes involved in the biological handling of excess cytosolic ferrous iron [12,13,14,15,16]. In particular, the study of *Drosophila melanogaster* ferritins has informed the field of insect iron physiology (reviewed in [17,18,19]). With exception of the testis-specific mitochondrial ferritin [20], most cell types of *Drosophila melanogaster* involved in iron storage accumulate ferritin in their endomembrane system [21,22,23,24,25]. Subcellular localization within the vesicular system comes with distinct evolutionary adaptations for the insect ferritins. First, the Ferritin 1 Heavy Chain Homolog (Fer1HCH) and Ferritin 2 Light Chain Homolog (Fer2LCH) subunits have N-terminal signal peptides that direct them to the endoplasmic reticulum [26,27]; Second, Fer1HCH and Fer2LCH are cross-linked to each other by disulfide bonds, giving rise to a highly organized symmetrical arrangement of 12 Fer1HCH and 12 Fer2LCH subunits in the assembled ferritin complex [28]; Third, the *Fer1HCH* and *Fer2LCH* genes share common enhancers (they are transcriptionally co-regulated) being chromosomal neighbors and also showing post-transcriptional co-regulation to ensure the provision of roughly equal amounts of subunits [16,21,29]. These regulatory relationships are conserved in other insects besides *Drosophila melanogaster* [30,31]; Fourth, iron loading into ferritin critically depends on transport from the cytosol to the endoplasmic reticulum, a function likely mediated by the zinc regulated and iron regulated transporter 13 (Zip13) [25]; Fifth, the two subunits Fer1HCH and Fer2LCH have been detected in distinct vesicular compartments at the initial stages of the ferritin biosynthetic process, one hour post-feeding on iron-containing media, suggesting that subunit-specific trafficking and post-translational modifications may precede the formation of the ferritin complex [21]. A recent complementary effort in mosquito cells is likely to provide independent information for the ferritin assembly and secretion processes [32].

Despite the differences between the subcellular accumulation of ferritin: in the cytosol of vertebrates [33], in the chloroplasts of plants [34] and in the secretory pathway of many insect ferritins (for insects with cytosolic ferritins see [4] and also the ferritin sequences of *Rhodnius prolixus* [35]), strong evolutionary links exist between ferritins from prokaryotes and archaea to eukaryotes [36,37,38,39,40]. In particular, the mechanism of iron mineralization in assembled ferritins is highly conserved [38,39,40]. Ferritin assembly is generally thought to occur spontaneously, aided by the high stability of the ferritin subunit dimers [41,42,43,44,45]. Recently, self-assembly of ferritin was shown to be required for achieving ferroxidase catalytic activity [46]. Given that ferritins isolated from different mammalian tissues show differences in the ratios of the two types of their subunits, the regulation of ferritin assembly *in vivo* requires further investigation [47,48,49,50,51,52,53].

The *Drosophila* intestine is highly compartmentalized with small groups of enterocytes and adjacent enteroendocrine cells specializing in different functions [54,55,56,57,58,59], including metal storage and detoxification [17,18,60,61,62,63,64]. The larval anterior midgut provides an ideal epithelium to observe the ferritin biosynthetic process because it contains large enterocytes, which do not normally express ferritin, but readily induce its expression upon iron treatment [2,16,21,22,65,66,67]. The *Fer1HCH*^G188^ allele, which splices the green fluorescent protein (GFP) into the endogenous *Fer1HCH* gene and assembles GFP-Fer1HCH subunits in iron-loaded ferritin complexes, was previously used together with Fer2LCH-specific antibodies to detect both subunits in larval intestines [21]. In the iron region, Fer2LCH subunits fully co-localized with GFP-Fer1HCH, *i.e.*, there were no vesicles in which the subunits could be seen separately [21]. These vesicles represent a specialized Golgi compartment, packed with assembled, iron-loaded ferritin [2,3]. In the anterior midgut, ferritin assembly had not occurred 1 h after the transfer of larvae on an iron-rich diet, but was complete by 4 h [21]. Accordingly, 1 h after the transfer, Fer2LCH was readily detectable in a separate vesicular compartment to GFP-Fer1HCH, whereas 4 h after the transfer only vesicles containing both subunits were detected in anterior midgut cells of *Fer1HCH^G^*^188/+^ larvae [21]. These observations led to a model, whereby individual ferritin subunits are modified in separate vesicular compartments prior to assembly of the ferritin complex. The present study was undertaken to further test the hypothesis of a regulated ferritin assembly process involving separate vesicular compartments by using fluorescent-protein-based imaging to allow for the simultaneous visualization of Fer1HCH and Fer2LCH subunits in the larval intestine.

## 2. Results and Discussion

To visualize the ferritin assembly process *in vivo*, a *UAS-mCherry-Fer2LCH* construct was designed. The mCherry fluorescent protein was inserted in the N-terminus of the *Fer2LCH* gene, immediately after the predicted cleavage site associated with the signal peptide that targets Fer2LCH to the endoplasmic reticulum [27]. To express mCherry-Fer2LCH in an iron-inducible manner in the larval anterior midgut, a *Fer2LCH-Gal4* driver was generated by transposition [68] of the *P{GawB}* element [69] into *Fer2LCH^EP^*^1059^ [10]. Both the parental EP and the new Gal4 lines were homozygous lethal, because normal *Fer2LCH* gene function was interrupted by the insertions. In contrast, *Fer2LCH-Gal4*, *UAS-Fer2LCH* recombinants were homozygous viable, indicating that the new driver could express heterologous *Fer2LCH* where it was required during development. *Fer2LCH-Gal4*, *UAS-mCherry-Fer2LCH* flies were not homozygous viable, consistent with previous observations that ferritin consisting solely of GFP-Fer1HCH and Fer2LCH subunits was not functional [10,21]. It was still possible, however, to form functional ferritin complexes if GFP-Fer1HCH was present together with Fer1HCH and Fer2LCH [21], which provided a rational to work with *UAS-mCherry-Fer2LCH* in the presence of endogenous *Fer2LCH*.

Two further *Fer2LCH-Gal4* lines became available from the Kyoto stock center [70] and all three lines gave identical intestinal expression. Ferritin is also expressed in the brain [10,24,71,72,73,74,75]. Images obtained from the brains indicated some differences between the three *Fer2LCH-Gal4* lines, but these results are not presented here.

### 2.1. Ferritin Gal4 Driver Lines Recapitulate Iron-Dependent Induction in the Anterior Midgut

**Figure 1:**
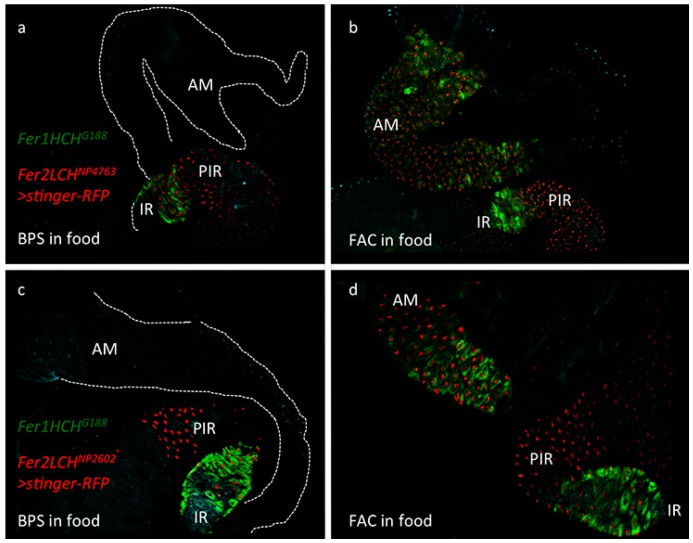
(**a**) Expression pattern of the *Fer2LCH^NP^*^4763^*-Gal4* driver line in the larval intestine as revealed by the nuclear stinger red fluorescent protein originating from *UAS-stinger-RFP*. Larvae were raised on a diet containing 200 µM of the iron chelator Bathophenanthroline Sulfate (BPS). Note that all cells of the iron region (IR), marked by Green Fluorescent Protein tagged Ferritin 1 Heavy Chain Homolog (GFP-Fer1HCH) from the *Fer1HCH^G^*^188^ allele, also express stinger-RFP, but the Gal4 driver expresses in cells posterior to the iron region (PIR) as well. The anterior midgut (AM) is marked with a dotted line in the top part of the panel; (**b**) The same genotype *Fer1HCH*^G188^/*Fer2LCH*^NP4763^, *UAS-stinger-RFP* raised on a diet supplemented with 1 mM Ferric Ammonium Citrate (FAC). Note the clear coincidence in anterior midgut cells of red fluorescence in the nuclei and green fluorescence in the cytoplasm. This region specifically responds to iron by expressing ferritin and the *Fer2LCH*^NP4763^*-Gal4* driver faithfully recapitulates the endogenous enhancer in this region of the intestine; (**c**) An identical pattern of expression for ferritin could be seen with the independent *Fer2LCH*^NP2602^*-Gal4* driver line in BPS treated larvae (**d**) and in the FAC treatment.

To test whether the *Fer2LCH-Gal4* lines recapitulated the endogenous ferritin expression pattern in larvae [22] and, in particular, the iron-dependent inducible expression in the anterior midgut, they were crossed to flies carrying a recombinant *Fer1HCH*^G188^, *UAS-stinger-RFP* chromosome. Simultaneous monitoring of cytoplasmic green fluorescence from the endogenous GFP-Fer1HCH protein trap and nuclear red fluorescence from cells expressing *Fer2LCH-Gal4* was possible in the progeny of this cross. Under iron limiting conditions, defined by addition of 200 µM Bathophenanthroline Sulfate (BPS; an effective iron chelator [15,20,76]) into the standard yeast and molasses based diet [77], *Fer2LCH^NP^*^4763^*-Gal4* expressed strongly in the iron region enterocytes, but also in cells posterior to this region (Figure 1a). Under dietary iron supplementation (1 mM Ferric Ammonium Citrate; FAC), the driver was clearly induced in the anterior midgut cells, in each and every cell that also expressed *GFP-Fer1HCH* from the endogenous gene promoter (Figure 1b). Expression in the iron region and in cells posterior to it remained. The same results were obtained with another driver, *Fer2LCH^NP^*^2602^*-Gal4* (Figure 1c,d). Thus, in the anterior midgut region, the *Fer2LCH-Gal4* lines recapitulated the well-established, iron-dependent ferritin expression pattern.

### 2.2. mCherry-Tagged Fer2LCH Subunit Expression Driven by Fer2LCH-Gal4 in the Intestine

**Figure 2:**
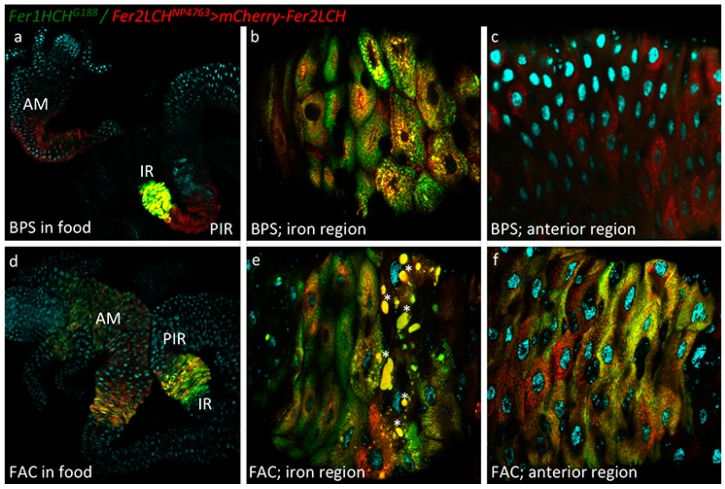
(**a**) Larvae of the genotype *Fer1HCH*^G188^*/Fer2LCH*^NP4763^, *UAS-mCherry-Fer2LCH* were grown on a diet supplemented with 200 µM BPS. Intestines were dissected, mounted in Vectashield with DAPI and imaged by confocal microscopy. Green fluorescence is from GFP-Fer1HCH; red fluorescence from mCherry-Fer2LCH; cyan fluorescence from DAPI. Using the 10× objective, GFP-Fer1HCH is readily observed only in the iron region (IR) as previously described. In contrast, mCherry-Fer2LCH is detected both in the iron region and in cells posterior to the iron region (PIR), recapitulating the expression pattern seen in Figure 1a, but it is also readily observable in the anterior midgut (AM); (**b**) Closer view of the iron region using the 40× objective (anterior is to the left) (**c**) and of the anterior midgut: only mCherry-Fer2LCH was detected here; (**d**) Larvae of the genotype *Fer1HCH*^G188^/*Fer2LCH*^NP4763^, *UAS-mCherry-Fer2LCH* were grown on a diet supplemented with 1 mM FAC. There is a visible induction of GFP-Fer1HCH and mCherry-Fer2LCH in the anterior midgut. In the majority of larvae observed (*n* > 10) the cells posterior to the iron region no longer express mCherry-Fer2LCH when raised on an iron-rich diet; (**e**) Closer view of the iron region—stars mark abnormally large vesicular compartments, which may represent an autophagic response in some cells of the larvae grown on food supplemented with 1 mM FAC; (**f**) Closer view of the anterior midgut region.

The intestines of 3rd instar larvae from the *Fer2LCH-Gal4*, *UAS-mCherry-Fer2LCH*/*Fer1HCH*^G188^ genotype raised in diets containing 200 µM BPS (Figure 2a–c) or 1 mM FAC (Figure 2d–f) were imaged to detect mCherry-tagged Fer2LCH subunit expression driven by *Fer2LCH-Gal4*. Under low iron conditions, mCherry-Fer2LCH accumulated in the iron region enterocytes (Figure 2a,b) and in cells posterior to the iron region (Figure 2a). Somewhat surprisingly, given the very low expression of *UAS-stinger-RFP* in the anterior midgut (Figure 1a,c), mCherry-Fer2LCH also accumulated in cells of the anterior midgut (Figure 2c). One possible explanation would be that mCherry-Fer2LCH is more stable than stinger-RFP in these cells and the fluorescence reflects an earlier or lower-level induction of the *Fer2LCH-Gal4* driver, or, alternatively, secreted mCherry-Fer2LCH is taken up by these cells, as has been shown to be the case for the nephrocyte-like garland cells [10].

When the intestines were dissected from larvae grown in diets supplemented with 1 mM FAC, both mCherry-Fer2LCH and GFP-Fer1HCH were detected in the anterior midgut region, but, curiously, mCherry-Fer2LCH appeared to be absent from the cells posterior to the iron region and only accumulated in the iron region enterocytes in the middle midgut (Figure 2d). This raised the question whether mCherry-Fer2LCH was being secreted to the hemolymph or to its neighboring iron-region cells or, less intuitively, whether it was being degraded despite the presence of dietary iron. The absence of mCherry-Fer2LCH is consistent with the known fact that these cells posterior to the iron region do not accumulate assembled, iron-loaded ferritin [22].

The reasons that would explain the differences in some cell types between the presence of the reporter gene expression and the mCherry-Fer2LCH accumulation are not understood, however these observations suggest that active transport of the ferritin subunits may be implicated in the assembly of functional ferritin complexes *in vivo*. Further evidence in support of this notion came from the altered accumulation of GFP-Fer1HCH (arising from *Fer1HCH*^G188/+^) when the secretory pathway was blocked in embryos by means of a lethal mutation in *Sec23* [10]. Nevertheless, *Fer2LCH-Gal4*, *UAS-mCherry-Fer2LCH*/*Fer1HCH*^G188^ larvae grown in 1 mM FAC accumulated mCherry-Fer2LCH in the same cell types where GFP-Fer1HCH was present (Figure 2e,f), suggesting that some aspects of the expected intestinal response to dietary iron were being reported faithfully with these tools.

Inspection of the iron region in the *Fer2LCH-Gal4*, *UAS-mCherry-Fer2LCH*/*Fer1HCH*^G188^ larvae revealed some abnormally large vesicular compartments, reminiscent of autophagosomes [78,79], where red and green fluorescence was readily observable. These compartments were substantially larger in intestines from larvae grown in 1 mM FAC food (compare Figure 2b–e) and they appeared to be present in the posterior half of the iron region. These larger compartments (autophagosomes) were not readily observable in intestines dissected from *Fer1HCH*^G188/+^ larvae and we therefore considered that they indicated a cellular stress imposed in the presence of mCherry-Fer2LCH and iron. The autophagosomes are a likely response to endoplasmic reticulum stress [80,81,82]. Moreover, it is possible that the fluorescent proteins are more resistant to degradation in this environment than their attached subunits [83], so the fact that mCherry and GFP signals are abundant suggests that both ferritin subunits had reached these compartments, but whether they were assembled, present as single subunits, or degraded remains unclear.

### 2.3. Subcellular Distribution of GFP-Fer1HCH and mCherry-Fer2LCH in Iron Region and Anterior Midgut Enterocytes

**Figure 3:**
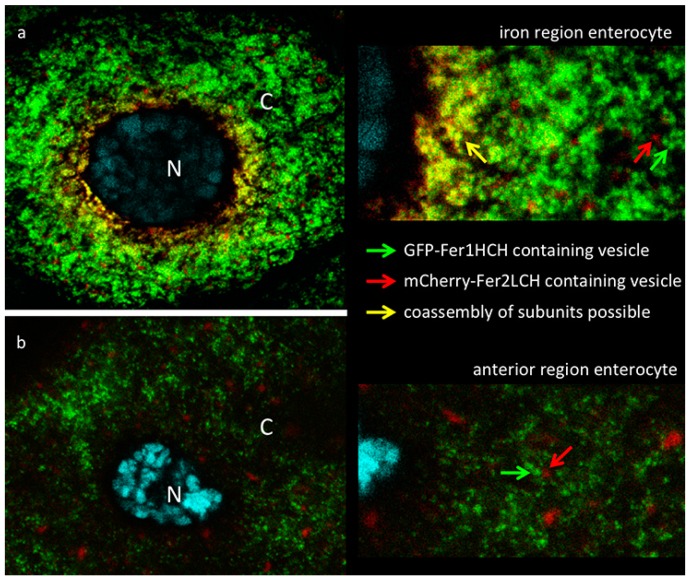
(**a**) Confocal image of iron region enterocyte (N—nucleus, DNA marked with DAPI in cyan, C—cytoplasm). Green fluorescence is from GFP-Fer1HCH; red fluorescence from mCherry-Fer2LCH; yellow color indicates overlap of signals. Larvae of the genotype *Fer1HCH*^G188^*/Fer2LCH*^NP4763^, *UAS-mCherry-Fer2LCH* were grown on a diet supplemented with 1 mM FAC. A close-up view on the right is provided for the viewer to note (i) that in a limited number of perinuclear Golgi vesicles (yellow arrow) GFP-Fer1HCH co-localizes with mCherry-Fer2LCH; and (ii) further in the periphery the vesicles containing GFP-Fer1HCH (green arrow) are clearly distinguishable from those containing mCherry-Fer2LCH (red arrow); (**b**) Single enterocyte of the anterior midgut region of the same larva—no co-localization observed between GFP-Fer1HCH and mCherry-Fer2LCH.

The cells that had no signs of autophagosome formation were imaged at a higher magnification (using a 63× objective & 2× optical zoom at the Confocal) to detect the subcellular localization of the ferritin subunits in enterocytes of *Fer2LCH-Gal4*, *UAS-mCherry-Fer2LCH*/*Fer1HCH*^G188^ larval intestines, raised in a diet supplemented with 1 mM FAC. Initial focus was on the iron region enterocytes (Figure 3a), where a perfect co-localization between mCherry-Fer2LCH and GFP-Fer1HCH had been expected [21]. In contrast to our expectations, only a limited number of vesicles containing both tagged ferritin subunits were visible and these were almost exclusively in the perinuclear region of cells. Further to the periphery, mCherry-Fer2LCH and GFP-Fer1HCH could be clearly detected in distinct vesicular compartments. Judging by morphological criteria and relative abundance, GFP-Fer1HCH was present in the Golgi-like vesicles that specialize in iron storage in these cells, whereas mCherry-Fer2LCH accumulated in a less abundant type of vesicle, which is normally devoid of ferritin (compare to Figure 6C in [21]). This distribution brought to question whether the mCherry-Fer2LCH subunits were being properly incorporated into the ferritin complexes of these cells.

Upon imaging the anterior midgut, co-localization within cells between GFP-Fer1HCH and mCherry-Fer2LCH was rare. A typical enterocyte in the anterior midgut is depicted (Figure 3b). Despite the ferritin induction as a response to iron, these cells accumulate mCherry-Fer2LCH and GFP-Fer1HCH in separate compartments. These results suggested that the mCherry-Fer2LCH subunits were not being incorporated into functional ferritin complexes. To directly observe the assembled ferritin complexes and the loading of iron into these, protein extracts from fly genotypes expressing GFP-Fer1HCH or mCherry-Fer2LCH under non-reducing SDS-PAGE were ran and the gels were stained for protein or iron, respectively.

### 2.4. Iron Loading in Ferritins with GFP-Fer1HCH Subunits Only Occurs When They Are Expressed from Fer1HCH^G*188*^ But not from Fer2LCH-Gal4, UAS-GFP-Fer1HCH Flies

**Figure 4:**
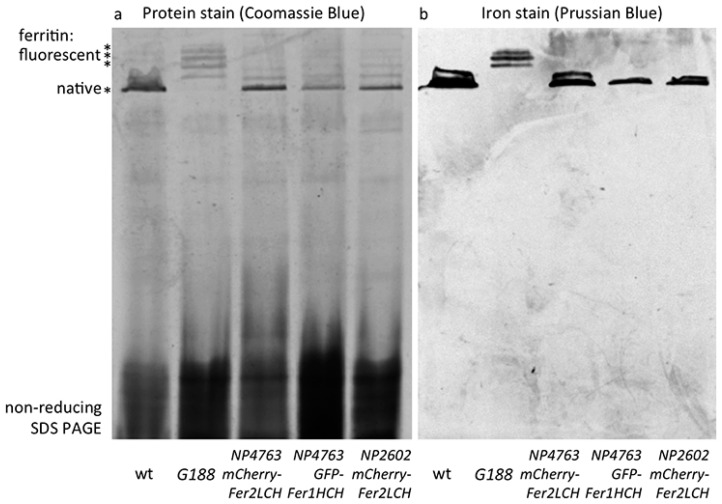
(**a**) Coomassie blue staining following non-reducing SDS PAGE of whole-fly homogenates of the indicated genotypes raised on 1 mM FAC supplemented food. The major high molecular weight band in these extracts (indicated by the single asterisk) is the native ferritin [15]. Higher molecular weight bands (indicated by three asterisks) represent assembled ferritin complexes with an increasing number of fluorescent protein subunits attached [21]; (**b**) Prussian blue staining to reveal iron-loaded ferritin molecules. Note that no native iron-loaded ferritin is detected in samples from *Fer1HCH*^G188/+^ fly homogenates, suggesting that in this genotype the ferritin assembly process efficiently combines GFP-Fer1HCH subunits with its endogenous Fer1HCH and Fer2LCH counterparts. In contrast, when mCherry-Fer2LCH subunit (lanes 3 and 5) or GFP-Fer1HCH subunit (lane 4) expression are driven by *Fer2LCH-Gal4*, only ferritin comprised from wild type subunits is iron-loaded.

Wild type ferritin and ferritin with a varying number of GFP-Fer1HCH subunits attached to the assembled complex (of 12 Fer2LCH:*x* Fer1HCH:*y* GFP-Fer1HCH subunits, where *x* + *y* = 12) have been previously analyzed by non-reducing SDS-PAGE and radioactive iron incorporation assays [21]. Ferritin iron is sufficiently concentrated as to be also readily observable with a simple incubation with potassium ferrocyanide in acid conditions (Prussian blue stain) and ferritin protein is the dominant abundant high molecular protein observed with Coomassie blue staining in extracts from adult flies analyzed in this manner [15,16,84]. Hence the first two lanes in Figure 4 represent the wild type control (with a prominent ferritin band representing the complex of 12 Fer1HCH and 12 Fer2LCH subunits) and the GFP-tagged ferritin from *Fer1HCH*^G188/+^, where wild type ferritin complexes are absent and new higher molecular weight complexes appear (representing increasing numbers of GFP-Fer1HCH subunits incorporated). Iron is accumulated in these *Fer1HCH*^G188/+^-specific ferritins.

When the *Fer2LCH-Gal4*, *UAS-mCherry-Fer2LCH* chromosome was tested (over a balancer chromosome, *i.e.*, in conditions where one copy of *Fer2LCH* was unaffected and both copies of *Fer1HCH* were present), higher molecular ferritin complexes appeared in the protein stains of gels, albeit in less abundance compared to the *Fer1HCH^G^*^188*/+*^ genotype (Figure 4a), suggesting that assembled ferritin complexes were present. However, the most abundant species was the wild type ferritin. Importantly, it was only in wild type ferritin that iron could be detected in these flies (Figure 4b). These results were consistent with some limited ferritin complex formation (*i.e.*, see Figure 3a) and with a more general conclusion that most functional (*i.e.*, iron-loaded) ferritin in these animals had not incorporated the mCherry-Fer2LCH subunit.

One remaining concern was whether the attachment of mCherry to Fer2LCH is the main reason behind these phenomena, for example by affecting the process of iron loading into ferritin. To test this idea, *UAS-GFP-Fer1HCH* transgenic flies were generated, whereby GFP was attached exactly at the same position as it is found in the *Fer1HCH*^G188^ protein trap allele and crossed them to *Fer2LCH-Gal4.* It was reasoned that the presence of a few GFP-Fer1HCH subunits in the assembled ferritin complex should not inhibit iron loading, given the positive control (*i.e.*, the *Fer1HCH*^G188/+^ genotype). Nevertheless, the *Fer2LCH-Gal4*, *UAS-GFP-Fer1HCH* flies were unable to produce detectable quantities of iron-loaded ferritin complexes containing GFP-Fer1HCH subunits. This genotype accumulated iron in ferritin complexes consisting exclusively of endogenous Fer1HCH and Fer2LCH subunits (Figure 4). To explain these observations, a hypothesis that the timing of *GFP-Fer1HCH* subunit expression determines whether ferritin iron loading occurs in GFP-Fer1HCH-containing ferritin complexes was proposed and tested.

### 2.5. A Model for Ferritin Biosynthesis in Anterior Midgut Enterocytes

**Figure 5:**
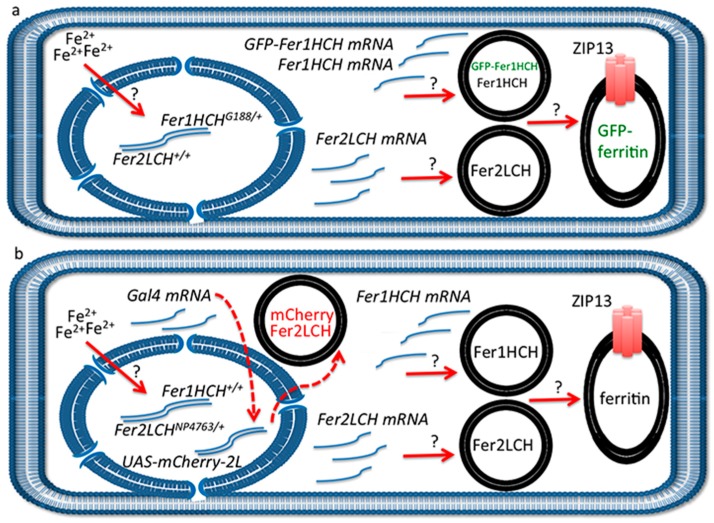
(**a**) Schematic representation of anterior midgut enterocyte from *Fer1HCH^G^*^188*/+*^ larvae at one hour post-feeding on 1 mM FAC. Iron has been sensed by an unknown mechanism in the cytosol, ferritin transcription has been induced (the transcription factors involved have not been experimentally determined [13,29]) and two types of vesicles have formed: one containing Fer2LCH subunits only and another containing Fer1HCH and GFP-Fer1HCH subunits. These vesicles will soon give rise to assembled, iron-loaded ferritin in a single type of Golgi vesicle (see [21] for evidence). The ZIP13 transporter is implicated in iron transport to the vesicles [25]. Question marks above the red arrows indicate that these processes are poorly understood; (**b**) Similar representation from *Fer2LCH-Gal4*, *UAS-mCherry-Fer2LCH* larvae. Again, iron has been sensed in the cytosol, ferritin transcription has been induced and two types of vesicles have formed: one containing Fer2LCH subunits only and the other containing Fer1HCH subunits only. There has also been synthesis of the transcription factor Gal4, which will move into the nucleus. When ferritin assembly and iron loading take place, there is no mCherry-Fer2LCH present. This model implies that approximately one hour later when mCherry-Fer2LCH will be synthesized from the action of the Gal4-UAS system (red dotted arrows), there will either be no remaining Fer1HCH-containing vesicles with which to co-assemble or the iron loading process on assembled ferritin has finished. The model further implies feedback inhibition of ferritin synthesis, resulting in a coordinated pulse of expression of both genes encoding for the ferritin subunits upon cellular iron entry.

The proposal is that cellular iron entry induces both ferritin subunits in a pulse, *i.e.*, *Fer1HCH* and *Fer2LCH* mRNAs are produced in a coordinated, non-continuous manner and that following their translation they are first processed separately, but then assembled rapidly, first as heterodimers [28], then into the complex that receives the excess iron (Figure 5a). Zip13 is required for the iron-loading step [25]. In addition, the presence of a ferritin subunit in the absence of its partner is not sufficient for complex formation. Indeed, previous studies have shown that heterozygous mutants (or RNA interference [16]) in either *Fer1HCH* or *Fer2LCH* produce half the amount of ferritin [21]. Similarly, overexpression experiments suggest that both ferritin subunits need to be induced to achieve a demonstrable increase in ferritin accumulation [21,24]. The recent discovery in the dipteran fly *Bactrocera dorsalis* of an alternatively spliced intron in *Fer2LCH* that leads to the insertion of a premature codon revealed a further aspect of the co-regulation of the two ferritin subunits, connecting transcriptional to post-transcriptional control [31].

An interpretation of the experimental results is depicted in Figure 5. According to our hypothesis, the reason for not seeing significant ferritin complex formation incorporating GFP-Fer1HCH or mCherry-Fer2LCH subunits when driven with the Gal4-UAS system is that they are produced too late in the timeframe of events that follow cellular iron entry. In other words, at the time endogenous Fer1HCH and Fer2LCH are being produced and processed, *Fer2LCH-Gal4* has induced the Gal4 transcription factor, but Gal4-induced transcription has not yet occurred (red dotted arrow in Figure 5b). At a later stage, when *UAS-mCherry-Fer2LCH* is expressed and translated, there are few Fer1HCH subunits available to form the ferritin complex; hence mCherry-Fer2LCH accumulates in a separate vesicle. The time-delay described here is inherent in the mode of action of the Gal4-UAS system [85], a drawback previously recognized and leading to the development of protein-trap systems [86,87,88]. Our model also accounts for the observation that *Fer2LCH-Gal4*, *UAS-Fer2LCH* flies are homozygous viable (the homozygous *Fer2LCH-Gal4* driver is lethal because the P-element insertion interrupts endogenous *Fer2LCH* function). In homozygous *Fer2LCH-Gal4*, *UAS-Fer2LCH* flies there will be no endogenous Fer2LCH subunits to complex with Fer1HCH at the time of cellular iron entry; therefore recently made Fer1HCH will not be used up and the temporal delay is accommodated in this situation.

### 2.6. Evidence that mCherry-Fer2LCH Is Incorporated in Iron-Loaded Assembled Ferritin Complexes When Co-Expressed Simultaneously with Fer2LCH

**Figure 6:**
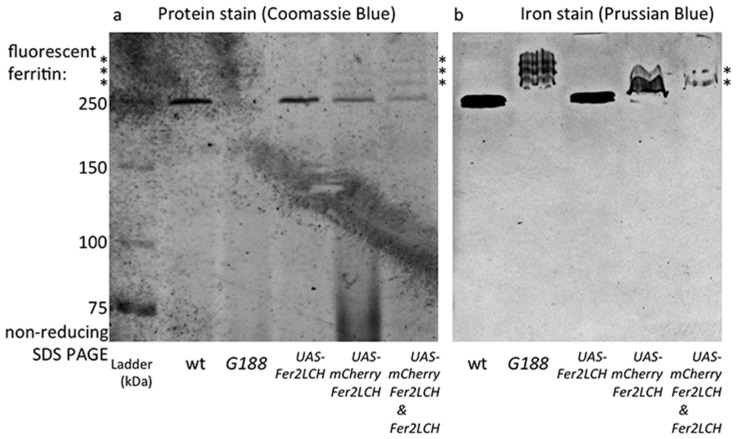
(**a**) Coomassie blue staining (**b**) and Prussian blue staining following non-reducing SDS PAGE of whole-fly homogenates (flies raised on 1 mM FAC) of the genotypes: wild type (wt); *Fer1HCH*^G188^^/+^; *Fer2LCH*^21BGal4^, *UAS-Fer2LCH/+*; *Fer2LCH*^NP4763^, *UAS-mCherry-Fer2LCH/+*; *Fer2LCH*^21BGal4^, *UAS-Fer2LCH/Fer2LCH*^NP4763^, *UAS-mCherry-Fer2LCH*. Asterisks denote tagged ferritins. Note the higher molecular weight, mCherry-tagged ferritins in samples from the *Fer2LCH*^21BGal4^, *UAS-Fer2LCH/Fer2LCH-Gal4*^NP4673^, *UAS-mCherry-Fer2LCH* genotype (last lane), suggesting that in this genotype mCherry-Fer2LCH subunits assemble with Fer2LCH and the endogenous Fer1HCH counterparts and the resulting ferritins become iron loaded. Asterisks denote ferritins assembled with fluorescent protein subunits.

To test the proposed model, *Fer2LCH-Gal4*, *UAS-Fer2LCH* was crossed to *Fer2LCH-Gal4*, *UAS-mCherry-Fer2LCH*, reasoning that in this way there would be no endogenous *Fer2LCH* expression (due to the Gal4 insertions), but Fer2LCH expressed from the *UAS* transgene would rescue and would be expressed at the same time with mCherry-Fer2LCH. Non-reducing SDS PAGE of whole-fly homogenates (from flies raised on 1 mM FAC) was performed and the gels were treated with Coomassie and Prussian blue stains (Figure 6). As predicted by the model, iron loading in ferritins assembled with mCherry-Fer2LCH was observed in the new genotype.

**Figure 7:**
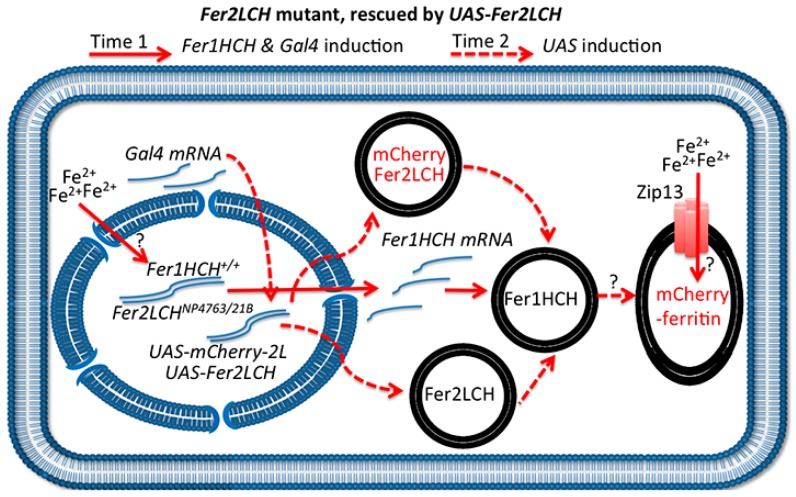
Schematic representation of the anterior midgut enterocyte from *Fer2LCH*^21BGal4^, *UAS-Fer2LCH/Fer2LCH-Gal4*^NP4673^, *UAS-mCherry-Fer2LCH* flies, fed on iron. The simultaneous (albeit delayed) expression of Fer2LCH and mCherry-Fer2LCH subunits in this genotype leads to the assembly and iron loading of mCherry-tagged ferritin. Solid red arrows indicate events taking place immediately after cytosolic iron sensing; dotted red arrows indicate subsequent Gal4/UAS dependent gene expression; question marks indicate that the detailed mechanisms of cellular iron sensing, ferritin assembly and iron loading remain unknown.

These results confirm that the mCherry-Fer2LCH subunit can in principle assemble with the Fer1HCH and Fer2LCH subunits giving rise to functional ferritin molecules. For these mCherry-tagged assembled ferritins to be iron-loaded, simultaneous timing of the expression of mCherry-Fer2LCH and Fer2LCH subunits is required (Figure 7). Nevertheless, iron loading was clearly less compared to the native ferritins. The same holds for GFP-Fer1HCH-containing ferritins [15,21]. Why this is the case is not presently understood, but the bulky tags may affect the folding of the subunits, resulting in diminished ferroxidase activity of the complex or interfering with iron delivery to ferritin.

### 2.7. New Tools Are Required for the in Vivo Imaging of Ferritin Assembly in the Drosophila Intestine

Cellular iron sensing is not yet understood in *Drosophila*, beyond the post-transcriptional Iron Regulatory Protein-Element paradigm [89]. A genetic screen designed to uncover the transcriptional factors involved in iron-induced transcription failed to reveal any, possibly because it only screened homozygous viable mutants [13]. Experiments presented here support the notion that the ferritin assembly is a highly regulated process, however more investigations are required to unravel the full sequence of events following cellular iron entry into the enterocytes of the anterior midgut. Generating a *Fer1HCH*^G188^, *Fer2LCH-Gal4* recombinant chromosome is an obvious yet challenging objective, as the two genes are direct chromosomal neighbors [29]. It would be helpful to obtain a fly strain expressing mCherry-Fer2LCH directly from the *Fer2LCH* promoter to support future studies. In this respect, the GFP-protein trap line *Fer2LCH*^CPTI100064^ [87] does not accumulate GFP-Fer2LCH in the intestines (data not shown). Our efforts to employ the P[acman] BAC libraries [90] to rescue ferritin deficiency mutants [10,15] were stalled by inefficient transgenesis of the 154,003 base pairs of the R22M06 BAC clone that includes the *Fer1HCH*, *Fer2LCH* genomic locus. Genetic engineering techniques in *Drosophila* are evolving at an incredible pace and a strategy for generating mCherry knock-in alleles in *Fer2LCH* using the Clustered Regularly Interspaced Short Palindromic Repeat associated technology can be considered [91,92,93]. Alternatively, the use of bisarsenic fluorescent probes, activated upon cage assembly, might be adopted by site-directed mutagenesis of *Fer1HCH* and *Fer2LCH* to generate optimal bisarsenic binding pockets and visualize the process *in vivo* [94,95]. This latter strategy, would come with the advantage of avoiding steric complications arising from the presence of the GFP and mCherry protein tags on the outside of the ferritin cage.

## 3. Materials and Methods

Wild type flies used in this study were collected in Tannes, Italy [8]. The *Fer1HCH*^G188^ allele has been characterized previously [10,15,21,22]. The Gal4 drivers *Fer2LCH*^NP2602^ and *Fer2LCH*^NP4763^ [70] were obtained from the Kyoto Stock Center (#104255 and #113517, respectively). *Fer2LCH*^21BGal4^ was generated by transposition [68] of the *P{GawB}* element [69] into *Fer2LCH*^EP1059^ and has been used before [10]. Tagged ferritin constructs *UAS-mCherry-Fer2LCH* and *UAS-GFP-Fer1HCH* were generated in the pCasper-UAST vector [69] by inserting, respectively, *mCherry* and *GFP* at the *N*-termini regions of the open reading frames for Fer2LCH and Fer1HCH, respectively, immediately following the predicted cleavage sites of the endoplasmic reticulum target sequences. GFP was inserted following aspartic acid 22 of Fer1HCH and mCherry following cysteine 23 of Fer2LCH.

The diet used in all experiments was based on yeast and molasses [77]. The addition of 200 µM BPS (final concentration) decreases ferritin and iron in the flies, whereas the addition of 1 mM FAC accumulates total body iron content and induces ferritin [10,15,21,22]. 3rd instar crawling larvae were selected immediately after the end of their feeding phase as they initiated foraging away from the fly food to the sides of the plastic vials in which they were reared. The larval cuticle was broken open, the internal organs were exposed but not dissected out; instead the samples were incubated in freshly prepared 4% paraformaldehyde and kept at −4 °C for 12 h. The next day, freshly prepared 4% paraformaldehyde was replaced for 2 h at room temperature, followed by three washes with phosphate saline buffer for 20 min each. Dissections were performed directly in PBS for *Drosophila* (Cold Spring Harbor Protocols) and the intestines were removed and mounted on Vectashield mounting medium containing DAPI. Imaging was performed at a Leica TCS SP8 confocal system coupled to a DMI6000 inverted microscope (Wetzlar, Germany).

Non-reducing SDS-PAGE was performed on 6% acrylamide gels, followed by Coomassie and Prussian blue stains, as described previously [15,84]. It is noted that the ferritin complex runs at higher apparent molecular weights in 8% and 10% acrylamide gels, but the resolution of the tagged ferritin complexes is less evident there.

## 4. Conclusions

Here, we described *Fer2LCH-Gal4* lines, which are iron-responsive in the anterior midgut region. These were used to drive *UAS-mCherry-Fer2LCH* and *UAS-GFP-Fer1HCH*. Ferritin complexes containing the mCherry-Fer2LCH or the GFP-Fer1HCH subunits induced in this way were, however, iron poor and iron was stored instead in ferritin complexes composed exclusively from the endogenous Fer1HCH and Fer2LCH subunits. This situation contrasts what is observed when *GFP* is directly spliced into the endogenous *Fer1HCH* transcript, as is the case in the *Fer1HCH*^G188/+^ genotype, where no ferritin complexes composed exclusively of Fer1HCH and Fer2LCH subunits were detected and iron was loaded instead to ferritin complexes assembling with GFP-Fer1HCH, endogenous Fer1HCH and endogenous Fer2LCH subunits.

From these findings, we conclude that the temporal delay inherent in the production of the Gal4 transcription factor and its movement to the nucleus to activate upstream sequences and produce tagged ferritin subunits impedes their incorporation into functional assembled ferritin complexes. We support this conclusion by showing that flies co-assemble iron loaded mCherry-tagged ferritin complexes when expression of *mCherry-Fer2LCH* is concurrent to that of *Fer2LCH*. Thus, ferritin assembly is a highly organized, temporally regulated, cellular process in *Drosophila*. Further experiments using alternative strategies are required to uncover the mechanistic details of insect ferritin assembly as it occurs *in vivo*.
